# Influence of Uncertainty and Surprise on Human Corticospinal Excitability during Preparation for Action

**DOI:** 10.1016/j.cub.2008.04.051

**Published:** 2008-05-20

**Authors:** Sven Bestmann, Lee M. Harrison, Felix Blankenburg, Rogier B. Mars, Patrick Haggard, Karl J. Friston, John C. Rothwell

**Affiliations:** 1 Sobell Department of Motor Neuroscience and Movement Disorders, Institute of Neurology, University College London, London WC1N3BG, United Kingdom; 2 Wellcome Trust Centre for Neuroimaging, Institute of Neurology, University College London, London WC1N3BG, United Kingdom; 3 Institute of Cognitive Neuroscience, University College London, London WC1N3BG, United Kingdom

**Keywords:** SYSNEURO

## Abstract

Actions are guided by prior sensory information [1–10], which is inherently uncertain. However, how the motor system is sculpted by trial-by-trial content of current sensory information remains largely unexplored. Previous work suggests that conditional probabilities, learned under a particular context, can be used preemptively to influence the output of the motor system [11–14]. To test this we used transcranial magnetic stimulation (TMS) to read out corticospinal excitability (CSE) during preparation for action in an instructed delay task [15, 16]. We systematically varied the uncertainty about an impending action by changing the validity of the instructive visual cue. We used two information-theoretic quantities to predict changes in CSE, prior to action, on a trial-by-trial basis: entropy (average uncertainty) and surprise (the stimulus-bound information conveyed by a visual cue) [17–19]. Our data show that during preparation for action, human CSE varies according to the entropy and surprise conveyed by visual events guiding action. CSE increases on trials with low entropy about the impending action and low surprise conveyed by an event. Commensurate effects were observed in reaction times. We suggest that motor output is biased according to contextual probabilities that are represented dynamically in the brain.

## Results and Discussion

A fundamental feature of human movement is that anticipatory knowledge of an impending action improves the speed and accuracy of responses. For example, reaction times (RTs) are faster when visual information indicates in advance which action we will have to make [11–14]. Sensory information provides useful cues for guiding actions, which may be probabilistic in nature. Learning the relative probabilities of impending actions may enable the nervous system to prepare motor output prior to an event.

Sensory cues that predict action enable a gradual build-up of preparatory activity in premotor and motor cortex prior to action [4–10, 20]. This build-up is reflected by specific excitability changes in corticospinal projections [15, 16]. Indeed, a growing number of studies demonstrate quantifiable effects of preparatory sensory information on the peripheral motor system [21, 22] and the spinal cord [21]. This implies that the predictive aspects of sensory information are learned and represented explicitly in the brain and that these representations influence action preparation at several levels [15, 16, 23–25].

**Figure 1:**
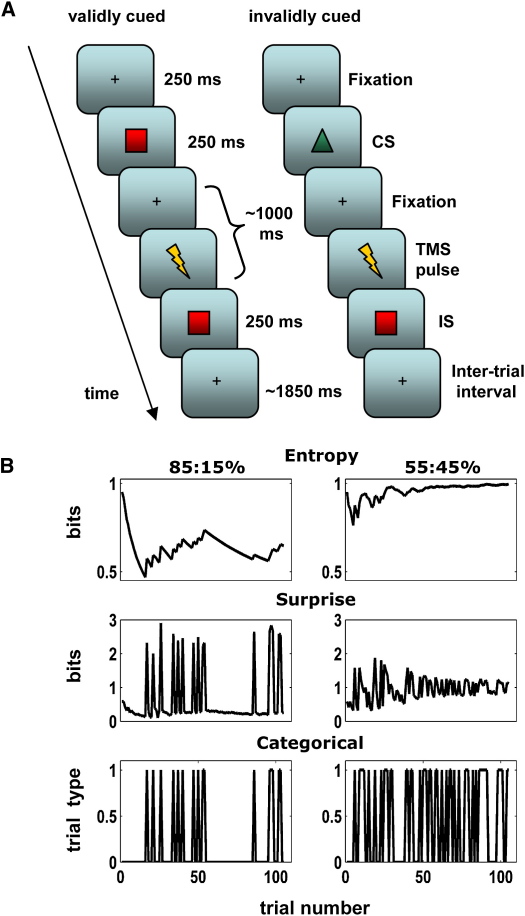
Experimental Task and Explanatory Variables (A) Schematic of the task. On valid trials, a preparatory CS predicted the identity of a subsequent IS, cueing a button press with the right thumb or little finger. On invalid trials the CS-IS mapping was invalid as the CS was followed by the alternative IS. The validity of the CS varied across blocks of 105 trials between 85:15%, 70:30%, and 55:45%, respectively, creating blocks with, low, medium, and high uncertainty about imperative stimuli. A single TMS pulse was applied during every trial, 200 ms before IS appearance. (B) Information theoretic and categorical quantities for two experimental blocks. Examples are shown of entropy and surprise during blocks with valid-invalid CS distributions of 85:15% (left panel) and 55:45% (right panel), respectively. Top panel, entropy; middle panel, surprise; and lower panel, regressors for a categorical model containing valid and invalid trial types. The ensuing time series were used as predictors for modeling CSE and RTs across the entire series of trials of each participant.

In the current study we asked how corticospinal excitability (CSE) changes when subjects prepare an action based on visual cues (Figure 1A) under changing degrees of uncertainty associated with an impending action. Understanding how the brain uses the predictability of events to inform preparation for action requires models of how this predictability is learned and represented over time rather than how they change on average. We, therefore, measured CSE prior to overt action by measuring muscular responses to stimulation of the motor cortex using transcranial magnetic stimulation (TMS) (see the Experimental Procedures and the Supplemental Experimental Procedures available online). We used established computational models to examine how the motor system might encode the probability of future events for action preparation.

Naïve, healthy participants prepared one of two actions (thumb or little-finger flexion) in an instructed delay task. In most trials a visual cue (CS) validly predicted a subsequent imperative stimulus (IS); on invalid trials, it was followed by the alternative imperative stimulus (Figure 1A). The proportion of valid cues (i.e., the predictability of required actions) was varied systematically across experimental blocks. Cue and imperative stimuli were sampled in each block from distributions containing 85%–15%, 70%–30%, or 55%–45% of valid-invalid trials. By using TMS, we probed effector-specific changes in CSE after the CS but before the IS signaled the required action (see the Experimental Procedures). An initial (conventional ANOVA) analysis of RTs revealed that subjects, on average, responded faster in blocks containing more valid trials (block type, F_2,18_ = 4.97; p < 0.01; Figure S1) and faster on valid versus invalid trials (trial type F_1,9_ = 6.9, p < 0.05). It, furthermore, suggested that participants learned the underlying conditional probabilities associated with each block (interaction trial-type × block-type; F_2,18_ = 7.23, p < 0.01). This interaction reflected, on average, faster responses when invalid cues were less likely (i.e., under less uncertainty). In line with these behavioral effects, variations of conditional probabilities and cue validity reliably affected CSE over both effectors. For both muscles CSE was larger in blocks with high predictability (thumb: F_2,18_ = 4.18, p < 0.05; little-finger: F_2,18_ = 9.56, p < 0.01); CSE also was larger for the cued versus uncued muscle (thumb: F_3,27_ = 6.38, p < 0.01; little-finger: F_3,27_ = 5.46, p < 0.01). For the thumb, trial type and block type interacted significantly (F_6,54_ = 3.91, p < 0.01); however, this failed to reach significance for CSE measurements of the little finger (F_6,54_ = 0.63, p = 0.7).

This confirms previous work that decisions depend on the probabilities of events and are, on average, represented in the motor system. However, the approach of comparing average activity levels between conditions does not disclose dynamic updating of representations about uncertainty that ensure efficient and fast action. We, thus, used two information-theoretic measures, surprise and entropy [17–19], to predict changes in CSE and RTs on a trial-by-trial basis under ideal observer assumptions. The “surprise” of a particular event (stimulus) is the improbability of it occurring. For example, in blocks containing 85% of valid cues, the probability of an invalidly cued (i.e., unexpected) stimulus is low and is more surprising than a validly cued IS. In contrast entropy is the average surprise over all possible outcomes and, therefore, is a measure of uncertainty about an event before it occurs. For example, consider the outcome of a coin toss. When the event – “heads” or “tails” – has an equal probability, then the overall uncertainty is maximal (i.e., entropy is greatest). Compare this to a biased coin in which probability of heads is 0.1 (and tails is 0.9). Here, entropy is lower (i.e., there is less overall uncertainty as to the outcome), but given the low probability of a head, the event of a head occurring is more surprising than for a fair coin. Surprise is event specific, whereas entropy pertains to the context in which that event occurs. This distinction is important as the entropy reflects uncertainty over all possible outcomes that could occur in a particular context (e.g., block); in contrast, surprise relates to a specific event (i.e., trial) and is an observation-bound quantity [18, 19] (Figure 1B).

Specifically, we quantified the conditional entropy, Ĥ, of an IS given a CS. This represents a rational measure of expected uncertainty (see [26] for discussion) under the assumption that contingencies among events are stationary (i.e., do not change over time). In addition we quantified the surprise, î, associated with an IS given a preceding CS. This latter quantity may be important for how we encode uncertainty, indicating that the stationary assumption has been violated. Because surprise can occur only after an event, its influence on CSE was modeled based on the surprise of the preceding trial.

**Figure 2:**
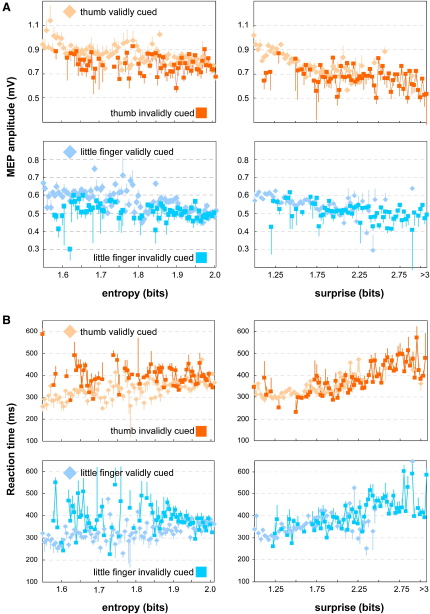
Influence of Entropy and Surprise on Reaction Times and Delay Period Corticospinal Excitability (A) CSE + SD for validly and invalidly cued trials from all subjects, plotted against entropy (left) and surprise (right). For display purposes data from all subjects were binned in steps of 0.005 (entropy) and 0.025 (surprise) bits, respectively. CSE was quantified from the peak-to-peak amplitude of motor-evoked potentials (MEP), elicited in the hand muscles contralateral to the TMS stimulation site. CSE was generally higher when uncertainty (entropy) was low, and trials were preceded by surprising events. (B) RTs + SD for validly and invalidly cued trials plotted against entropy (left) and surprise (right). For display purposes data from all subjects were binned in steps of 0.005 (entropy) and 0.025 (surprise) bits, respectively. Reaction times were generally faster when uncertainty (entropy) and surprise were low.

**Figure 3:**
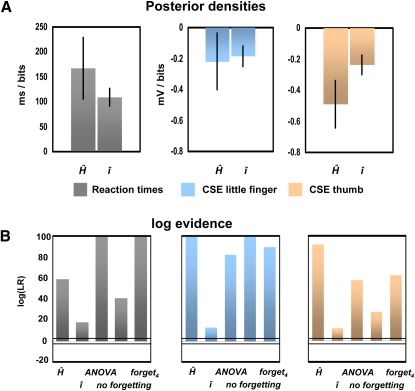
Modeling Results (A) Model predictions given by the posterior densities (mean ± SD) of weights of regressors from the model containing entropy and surprise. These represent a population effect (i.e., given data and a model over all subjects). For example, for RTs, these encode response time per bit of information presented to subjects. RTs (gray bars) increased with uncertainty, Ĥ, and surprise, î. Conversely, CSE (blue bars, little finger; red bars, thumb) decreased with entropy and surprise. Responses for each muscle were modeled for trials in which the corresponding imperative stimulus had occurred. (B) Model comparison. Bar-plot of the log-marginal likelihood ratio (i.e., approximate difference between the log evidence of two competing models) was used to compare models. Importantly, this includes a model complexity term. Positive values indicate more evidence for the model containing both entropy and surprise, whereas negative values indicate more evidence for one of the alternative models. For both outcome measures (RTs, gray; CSE, blue [little finger]; red [thumb]), we found substantially more evidence (ratio >3, indicated by the horizontal black lines) for the model accounting for both uncertainty and surprise. Abbreviations: Ĥ, entropy; î, surprise; ANOVA, conventional model comprising indicator variables identifying trial type; no forgetting (all trials over all blocks are taken into account); forget_4_, near maximal forgetting (only the previous four trials are taken into account).

Figure 2 shows that RTs and muscle-specific CSE changes are influenced by both entropy and surprise, although here we plot these data for validly and invalidly cued trials. In our analysis the information about block-type (which the subjects did not have explicit access to) is implicitly encoded by entropy and surprise computed from the cue and imperative stimulus presented during each trial. High entropy was associated with a small delay-period CSE (thumb, −0.49 mV/bit; little finger, −0.21 mV/bit). A surprising imperative stimulus on the preceding trial resulted in a similar decrease of CSE (thumb, −0.24 mV/bit; little finger, −0.19 mV/bit; see Figure 3A). Therefore, delay-period CSE of the cued muscle was lower when preparatory cues resolved less uncertainty (entropy) and when surprise induced by the preceding IS was large (Figure 3A). Concomitant changes were seen in participant's behavior who generally responded more slowly when entropy was high (167 ms/bit) and to surprising events (109 ms/bit).

We used Bayesian model comparison to compare different explanations of the data. These comprised three types of models. Firstly, we assumed that events were stationary and unchanging within a block. This matched the true generative distribution from which events (CS, IS) were sampled. In other words all previous blocks were forgotten in an optimal way and only trials within the current block were weighted equally. This assumption is ideal in relation to the actual experimental paradigm but assumes knowledge about the block structure of events. Moreover, in a more general setting, contingencies change slowly with a greater or lesser volatility [27]. We, therefore, included two alternative models based upon Bayesian observers with suboptimal forgetting. The first involved no forgetting and, so, included all trials over all blocks preceding the current trial. This is suboptimal because contingencies in fact changed from block to block. The second used only the four most recent trials, which we refer to as “fast forgetting”. Again this is suboptimal because this observer fails to accumulate evidence that is available within a block (see the Supplemental Experimental Procedures and Figure S2).

We used Bayesian model comparison to evaluate the evidence for these models. The intuition here is that the probability that data were generated by a model, i.e., the model evidence, can be approximated by the marginal likelihood of the data. This can be computed for two competing models and their ratio used as a measure of evidence in favor of one explanation, i.e., model. As model parameters are integrated out in this procedure, the model evidence includes a complexity term that penalizes models with more parameters, e.g., more columns in the design matrix in a general linear model. We report the log of this ratio of probabilities so that positive values >3 indicate evidence (20:1 odds) in favor of the model containing entropy (Ĥ) and surprise (î) [18]. As shown in Figure 3B, the important result here is that in all cases, evidence supported the model based on both entropy and surprise given the CSE and RT data. In other words for both outcome measures evidence was substantially larger for the model of both entropy and surprise compared to either entropy (Ĥ) or surprise (î) alone, a conventional (ANOVA) model comprising indicator variables identifying trial type, a model without forgetting, or a model with near maximal forgetting.

Our data suggest that the brain infers the probabilistic context of events, given past sensory information, in order to optimize and guide voluntary action. Although it has been suggested previously that the output of the motor system is, on average, influenced according to the past experience of ongoing sensory events [16], the present results show that this interaction can be predicted by using estimated quantities about uncertainty among events. This finding suggests that the brain tries to minimize prediction error [28, 29] by taking into account both the past history of events and the most recent experience. The influence of each can be computed and assigned with an information content that is then continuously channeled into motor regions to control the excitability of expected motor outputs. This may help to overcome inherent delays and noise in sensory feedback [1, 28], which inevitably slow response times.

Understanding how anticipatory presetting in the motor system is influenced by learning about, for example, the probabilistic context that currently guides our action requires formal models of this updating. Here, we explicitly modeled entropy and trial-by-trial surprise to predict preparatory set directly from the probabilistic structure of sensory events. Variations in delay-period CSE were indeed best explained by accounting for both entropy and surprise. Our analysis goes beyond previous studies about the importance of average reaction time [11–13, 30, 31] and CSE [15, 16] changes during preparation for action. These do not disclose whether CSE is reconfigured by predictions about events on a trial-by-trial basis nor reveal the relative contributions of entropy and surprise. We show the influence of these variables on the motor system during preparation for action and the advantage of our computationally informed model over categorical predictors that are not informed about the time-evolving estimates of probabilistic context. In the present case we achieved this under the assumption of an ideal observer. This approach provides a useful framework with minimal assumptions within which context can be modeled to predict corresponding changes in motor output and behavior.

Recently, Yu and Dayan introduced a distinction between expected and unexpected uncertainty [26]. Although these are heuristic concepts from the point of view of information theory, they can be associated with the distinction between entropy and surprise. In this context surprise corresponds to unexpected uncertainty that represents a violation of the expected uncertainty, represented by entropy. Moreover, had we modeled optimum forgetting or volatility [27] in our ideal Bayesian observer, unexpected uncertainty could be used to optimize the degree of forgetting. Taken together, several other modes of probabilistic context have been proposed [26, 27, 32, 33] and may prove useful for the study of preparatory delay period activity.

Previous neuroimaging work has suggested that entropy is encoded in the anterior hippocampus [19]. In contrast a bilateral parieto-fronto-thalamic network has been implicated in encoding surprise [19, 34]. The present results suggest that predictions about events based on both these variables can modulate the voluntary motor system on a trial-by-trial basis. Furthermore, the present findings show how measurements of corticospinal excitability with TMS can provide a window to examine computational processes about how humans implement decisions in real time. We conclude that motor output is flexibly shaped by contextual probabilities that are learned and represented dynamically in the brain.

## Experimental Procedures

### Participants

13 healthy right-handed volunteers (five females ages 27 ± 3.3 years) participated in the study. They received £10/hr monetary compensation for their participation. All participants had normal or corrected-to-normal visual acuity and reported no history of neuropsychiatric illness. Experiments were conducted according to the Declaration of Helsinki, with local ethics approval and informed consent. None of the participants reported any side effects of the experimental procedures. Participants were seated in a comfortable reclining chair with a mounted chin rest and nose bridge, which helped maintain head position. Three participants were excluded from analysis because of an excessive number of error trials (23.8%, 26.3%, and 33.4%, respectively).

### Behavioral Task

Participants performed an instructed delay task in which an arbitrary CS (green upward triangle or red ellipsoid) was followed by an IS (green upward triangle or red ellipsoid) presented approximately 1000 ms later (Figure 1A). The CS and IS were arbitrarily assigned to a right thumb or right little-finger button press, respectively, prior to the experiment. This stimulus-response mapping was balanced across participants. Instructions were given to respond as quickly and accurately as possible upon appearance of the IS. No performance feedback was given and participants were instructed to relax their hand muscles until the IS had been presented. Participants performed one practice block (105 trials) to ensure learning of the arbitrary stimulus-response mapping prior to the main experiment (see also the Supplemental Data). During the practice block the CS predicted the IS (and, hence, the required motor response) with 85% validity, and response feedback was provided. After this another practice block (105 trials) with TMS applied to the left M1 hand region was performed to ensure participants could perform the task comfortably in the presence of TMS. After a brief pause at the end of each block, the instructions were repeated.

In the main experiment the true CS validity varied across six blocks of 105 trials. Critically, the number of validly and invalidly cued trials in each block varied randomly across the experiment, containing either 85%:15%, 70%:30%, and 55%:45% of valid-invalid trials, respectively. The predictability of motor responses was, therefore, not simply stimulus bound but depended on the context established by preceding trials. Note that there was no underlying sequence governing stimulus presentation, only the relative proportions of stimuli were varied from block to block. No information about the underlying change in cue validity was given to participants at any time. Each validity condition was repeated twice in pseudorandom order (precluding back-to-back repetition of block types). The CS provided low, medium, or high uncertainty about which movement to make. Although the predictability of the required movement (based on the CS) was varied between blocks, each movement (thumb or little finger) was cued equally as often. The CS, therefore, validly (validly cued thumb, validly cued little finger) or invalidly (invalidly cued thumb, invalidly cued little finger) cued the required movement. After each block, a short pause of approximately 2 min was given.

By using single TMS pulses to the contralateral hand representation of left M1, CSE was probed 200 ms (±16 ms timing uncertainty) before the IS signaled which movement had to be performed (Figure 1A). This allowed us to assess the CSE in our subjects [35] before an overt movement was executed [15, 16]. Note that TMS was used to quantify CSE during the delay period [15, 16] before subjects knew which action they were going to make; it was not used to perturb their responses (as in virtual lesion studies). Although TMS pulses provided highly salient cues with respect to the time of IS presentation, they were entirely noninformative regarding its identity. Presentation of visual stimuli and synchronization with TMS was implemented by using MATLAB (The MathWorks, Natick, MA) custom stimulus presentation toolbox (http://www.vislab.ucl.ac.uk/Cogent2000/index.html).

### Data Analysis

Reaction times were calculated as the time between IS onset and the subsequent button press. Peak-to-peak MEP amplitudes were measured by using in-house software. Baseline EMG activity was calculated for each trial from the period between −500 and −200 ms prior to IS presentation. Trials were labeled as error trials with the following criteria: (1) RT of 80 ms or less, (2) omitted responses, (3) outlier RT and MEP responses as identified using Grubbs test (α = 0.05), and (4) baseline EMG activity exceeding 100 μV for more than 50 ms. Error trials (13.38% in total) were entered into the main modeling analysis as covariates of no interest. Repeated-measures ANOVA with factors trial type (thumb valid/invalid, little finger valid/invalid) and block type (85:15%, 70:30%, 55:45%) were used to assess their effect on RTs and MEPs.

### Computing Entropy and Surprise

In the text that follows we describe how to estimate the conditional probabilities needed to compute entropy (i.e., uncertainty) and surprise (see the Supplemental Experimental Procedures).

#### Multinomial and Dirichlet distributions

The notation here follows that used in [36]. Consider a discrete variable *x* that can take values from 1 to K. In our paradigm each trial comprises two stimuli, CS and IS, that induce four events: thumb validly cued, thumb invalidly cued, little finger validly cued, and little finger invalidly cued (i.e., K = 4). The vector $p=\left[p_{1},\ldots p_{K}\right]$ parameterizes probability distributions on *x*, where

(1) $$P\left(x=k\right)=p_{k}⇐P\left(x|p\right)=\prod_{k=1}^{K}p_{k}^{\delta \left(x=k\right)},$$

and δ(x=k) is an indicator function. This is a multinomial distribution where $p_{k}$ is the probability of each outcome pair or their joint probability, *p_ij_*, where *i* and *j* denote the IS and CS, respectively. The conditional probability of the *i*-th IS, given the *j*-th CS, is represented by $p_{i|j}=P\left(IS=i|CS=j\right)$ and relates to the joint distribution as follows:

(2) $$\begin{array}{ccc} \begin{array}{l} p_{1}=P\left(IS=\mathrm{thumb},CS=\mathrm{thumb}\right)\\ p_{2}=P\left(IS=\mathrm{finger},CS=\mathrm{thumb}\right)\\ p_{3}=P\left(IS=\mathrm{thumb},CS=\mathrm{finger}\right)\\ p_{4}=P\left(IS=\mathrm{finger},CS=\mathrm{finger}\right) \end{array} & \Rightarrow & \begin{array}{l} p_{\mathrm{thumb}|\mathrm{thumb}}=p_{1}/\left(p_{1}+p_{2}\right)\\ p_{\mathrm{finger}|\mathrm{thumb}}=p_{2}/\left(p_{1}+p_{2}\right)\\ p_{\mathrm{thumb}|\mathrm{finger}}=p_{3}/\left(p_{3}+p_{4}\right)\\ p_{\mathrm{finger}|\mathrm{finger}}=p_{4}/\left(p_{3}+p_{4}\right) \end{array} \end{array}.$$

Given $p_{i|j}$ we can compute the surprise about the imperative stimulus IS given the CS

(3) $$i\left(IS=i|CS=j\right)=-\log_{2}p_{i|j}.$$

Similarly, for each trial the conditional uncertainty, or entropy, about IS, given CS is simply

(4) $$H\left(IS|CS=j\right)=-\sum_{i}p_{i|j}\log_{2}p_{i|j}.$$

Entropy is a scalar quantity that reflects the average uncertainty about an event sampled from all possible events, whereas the surprise relates to the probability of a specific event and, so, is an observation-bound scalar quantity [18, 19] (Figure 1B).

These provide subject-specific explanatory variables (surprise, entropy) used in a hierarchical general linear model (GLM) whose parameters were optimized by using an empirical Bayes procedure (see the Supplemental Experimental Procedures). In this analysis we discarded any information about block-type (which the subjects did not have access to) but updated entropy and surprise on a trial-by-trial basis, based on what the subject actually observed (see Figure 1B and the Supplemental Experimental Procedures for details).

Note that the effects of surprise on RT pertained to the surprise of the imperative stimulus eliciting the response. In contrast, for the CSE data, given that the surprise of the IS cannot affect CSE in the same trial (because it was measured before the IS occurred), we used the surprise from the preceding trial.

## Supplemental Data

Document S1. Supplemental Experimental Procedures and Two Figures

## References

[1] Kording K.P., Wolpert D.M. (2006). Bayesian decision theory in sensorimotor control. Trends Cogn. Sci..

[2] Bays P.M., Wolpert D.M. (2007). Computational principles of sensorimotor control that minimize uncertainty and variability. J. Physiol..

[3] Carpenter R.H. (2004). Contrast, probability, and saccadic latency; evidence for independence of detection and decision. Curr. Biol..

[4] Roux S., MacKay W.A., Riehle A. (2006). The pre-movement component of motor cortical local field potentials reflects the level of expectancy. Behav. Brain Res..

[5] Wise S.P., Weinrich M., Mauritz K.H. (1983). Motor aspects of cue-related neuronal activity in premotor cortex of the rhesus monkey. Brain Res..

[6] Salinas E., Romo R. (1998). Conversion of sensory signals into motor commands in primary motor cortex. J. Neurosci..

[7] Tanji J., Evarts E.V. (1976). Anticipatory activity of motor cortex neurons in relation to direction of an intended movement. J. Neurophysiol..

[8] Bastian A., Riehle A., Erlhagen W., Schoner G. (1998). Prior information preshapes the population representation of movement direction in motor cortex. Neuroreport.

[9] Nakamura K. (2006). Neural representation of information measure in the primate premotor cortex. J. Neurophysiol..

[10] Crammond D.J., Kalaska J.F. (2000). Prior information in motor and premotor cortex: Activity during the delay period and effect on pre-movement activity. J. Neurophysiol..

[11] Hick W.E. (1952). On the rate of gain of information. Q. J. Exp. Psychol..

[12] Requin J., Granjon M. (1969). The effect of conditional probability of the response signal on the simple reaction time. Acta Psychol. (Amst.).

[13] Hyman R. (1953). Stimulus information as a determinant of reaction time. J. Exp. Psychol..

[14] Naatanen R. (1970). The diminishing time-uncertainty with the lapse of time after the warning signal in reaction-time experiments with varying fore-periods. Acta Psychol. (Amst.).

[15] Mars R.B., Bestmann S., Rothwell J.C., Haggard P. (2007). Effects of motor preparation and spatial attention on corticospinal excitability in a delayed-response paradigm. Exp. Brain Res..

[16] van Elswijk G., Kleine B.U., Overeem S., Stegeman D.F. (2007). Expectancy induces dynamic modulation of corticospinal excitability. J. Cogn. Neurosci..

[17] Shannon C.E. (1948). A mathematical theory of communication. Bell System Technical Journal.

[18] Harrison L.M., Duggins A., Friston K.J. (2006). Encoding uncertainty in the hippocampus. Neural Netw..

[19] Strange B.A., Duggins A., Penny W., Dolan R.J., Friston K.J. (2005). Information theory, novelty and hippocampal responses: Unpredicted or unpredictable?. Neural Netw..

[20] Rubino D., Robbins K.A., Hatsopoulos N.G. (2006). Propagating waves mediate information transfer in the motor cortex. Nat. Neurosci..

[21] Prut Y., Fetz E.E. (1999). Primate spinal interneurons show pre-movement instructed delay activity. Nature.

[22] Corneil B.D., Olivier E., Munoz D.P. (2004). Visual responses on neck muscles reveal selective gating that prevents express saccades. Neuron.

[23] Cisek P. (2006). Integrated neural processes for defining potential actions and deciding between them: A computational model. J. Neurosci..

[24] Cisek P. (2007). Cortical mechanisms of action selection: The affordance competition hypothesis. Philos. Trans. R. Soc. Lond. B Biol. Sci..

[25] Terao Y., Furubayashi T., Okabe S., Mochizuki H., Arai N., Kobayashi S., Ugawa Y. (2007). Modifying the cortical processing for motor preparation by repetitive transcranial magnetic stimulation. J. Cogn. Neurosci..

[26] Yu A.J., Dayan P. (2005). Uncertainty, neuromodulation, and attention. Neuron.

[27] Behrens T.E., Woolrich M.W., Walton M.E., Rushworth M.F. (2007). Learning the value of information in an uncertain world. Nat. Neurosci..

[28] Kording K.P., Wolpert D.M. (2006). Probabilistic mechanisms in sensorimotor control. Novartis Found. Symp..

[29] Friston K., Kilner J., Harrison L. (2006). A free energy principle for the brain. J. Physiol. (Paris).

[30] Niemi P., Naatanen R. (1981). Foreperiod and simple reaction-time. Psychol. Bull..

[31] Niemi P., Keskinen E. (1980). Visual stimulus-intensity and location probability - interactive effects on choice reaction-time. Scand. J. Psychol..

[32] Yang T., Shadlen M.N. (2007). Probabilistic reasoning by neurons. Nature.

[33] Carpenter R.H., Williams M.L. (1995). Neural computation of log likelihood in control of saccadic eye movements. Nature.

[34] Huettel S.A., Song A.W., McCarthy G. (2005). Decisions under uncertainty: Probabilistic context influences activation of prefrontal and parietal cortices. J. Neurosci..

[35] Di Lazzaro V., Oliviero A., Pilato F., Saturno E., Dileone M., Mazzone P., Insola A., Tonali P.A., Rothwell J.C. (2004). The physiological basis of transcranial motor cortex stimulation in conscious humans. Clin. Neurophysiol..

[36] Minka, T. Bayesian inference, entropy, and the multinomial distribution. http://research.microsoft.com/∼minka/papers/multinomial.html. 2003.

