# Supplementary material for: Influence of Uncertainty and Surprise on Human Corticospinal Excitability during Preparation for Action
Source: Curr Biol. 2008 May 20;18(10):775–80. doi: 10.1016/j.cub.2008.04.051 (PMC2387198; doi:10.1016/j.cub.2008.04.051)
Supplement: Document S1. Supplemental Experimental Procedures and Two Figures [file mmc1.pdf]

Supplemental Data

Influence of Uncertainty and Surprise on Human Corticospinal Excitability during Preparation for Action

Sven Bestmann, Lee M. Harrison, Felix Blankenburg, Rogier B. Mars, Patrick Haggard, Karl J. Friston, and John C. Rothwell

Supplemental Experimental Procedures

Experimental Setup and Stimuli

Arbitrary visual cues (CS) and imperative stimuli (IS) were presented for 250 ms (Figure 1A). After CS presentation, the IS was presented approximately 1000 ms later [± a random value sampled from a normal distribution $N(\mu = 0, \sigma^2 = 40)$]. The CS or IS did not provide any spatial information and were arbitrarily mapped onto required finger responses; CS and IS were arbitrarily assigned to a right thumb or right little finger button press, respectively, prior to the experiment. Visual stimuli (upward triangle or ellipsoid) were equated for surface area and subjectively matched for brightness in a behavioral pilot experiment. The mean intertrial interval was 1850 ms. Visual stimuli were presented centrally against a gray background on a 21 inch computer screen refreshed at 60 Hz at a viewing distance of 400 mm, subtending $\sim 4^\circ$ of visual angle. A small central cross ($\sim 0.5^\circ$ of visual angle) helped to maintain central fixation.

Transcranial Magnetic Stimulation and Electromyographic Recordings

TMS was applied using a 70 mm figure-of-eight coil connected to a Super Rapid stimulator (Magstim Co., Dyfed, Wales, UK). The site of stimulation was optimal for eliciting stable MEPs in both the right abductor pollicis brevis (APB, thumb) and abductor digiti minimi (ADM, little finger), approximately perpendicular to the central sulcus. Biphasic pulses of approximately 250 μs were applied with the current of the initial phase in antero-posterior direction. The coil position was marked on the volunteer's head and the TMS coil was held in constant position by using a two-joint coil holder (Manfrotto, Bassano del Grappa, Italy). Stimulation intensity was kept constant throughout the experiment and adjusted to evoke MEPs in both target muscles reliably (mean MEP amplitude across all experimental blocks: little finger, 0.62 ± 0.21 mV; thumb, 0.73 ± 0.33 mV). Note that although MEP amplitudes in both muscles usually differed in their peak-to-peak amplitude, we were interested in their relative changes and how they were modulated by entropy and surprise. The inter-TMS stimulus interval minimised carryover effects between trials and conformed to safety guidelines for TMS [S1]. Surface electromyographic (EMG) recordings were made using a belly-tendon montage with Ag/AgCl-plated surface electrodes (9 mm diameter). Raw EMG signal were amplified and filtered by using Digitimer D150 amplifiers (Digitimer Ltd., Welwyn Garden City, Herts., UK), with a time constant of 3 ms and a low-pass filter of 3 kHz. Signals were recorded via a CED 1401 laboratory interface (Cambridge Electronic Design Ltd., Cambridge, UK) and stored on a PC for later analysis using a sampling rate of 5 kHz.

As in previous work on delay-period CSE changes [S2–S6], we did not find evidence for condition-specific biases due to the TMS stimulus per se. When compared to our behavioral training session, we found a small yet significant reduction in reaction times relative to the corresponding TMS block of our main experiment (validly cued trials: 315 ± 10 ms (TMS main experiment) versus 329 ± 17 ms (behavioral training); $F_{1,9} = 6.12$, $p < 0.05$; invalidly cued trials: 403 ± 32 ms (TMS main experiment) versus 421 ± 27 ms (behavioral training); $F_{1,9} = 5.39$, $p < 0.05$). This is likely to be due to nonspecific intersensory facilitation arising from the sound generated upon TMS coil discharge. In addition, participants had to learn the arbitrary stimulus-response associations during the behavioral training session, additionally slowing average reaction times. This suggests that participants did not respond abnormally due to the mere presence of a TMS pulse during the preparatory delay period.

Model Specification and Estimation

To evaluate p_k, where $k = 1,\ldots,K$, we assume the subjects learn p_k as follows: The joint likelihood of $X = \{x_1, \ldots, x_N\}$ is

$$P(X|p) = \prod_{k=1}^{K} p_k^{N_k}, \tag{1}$$

where $N_k = \sum \delta(x_i = k)$ is the number of instances of the k-th trial-type. The conjugate prior of a multinomial is a Dirichlet distribution,

$$P(p|\alpha) \sim D(\alpha_1, \ldots, \alpha_K) = \frac{1}{Z(\alpha)} \prod_{k=1}^{K} p_k^{\alpha_k - 1}. \tag{2}$$

This has hyperparameters, $\alpha = [\alpha_1, \ldots, \alpha_K]^T$ and normalization constant $Z(\alpha)$. Given the likelihood and prior (Equations 1 and 2), we can compute the posterior distribution, which is also Dirichlet (because the prior is conjugate):

$$P(p|X,\alpha) \sim D(N_k + \alpha_k). \tag{3}$$

We assume the initial prior has hyperparameters, $\alpha_k = 1$, i.e., is uniform. This assumes that at the beginning of a block participants start with the prior that all events are equally likely.

Equation 3 gives the conditional density of the multinomial parameters we require. The expectation of event k given N observations is given by the predictive distribution,

$$p_k = \frac{N_k + \alpha_k}{N + \sum \alpha_k} = \frac{N_k + 1}{N + K}. \tag{4}$$

These values were computed sequentially throughout a block and entered into equations 3 and 4 from the main text. This modeling rests on ideal observer assumptions; several studies show that human observers can compute the predictability of sensory events and perform accordingly, close to the performance of an ideal observer [S7–S12]. Note also that in this analysis, we discard any information about trial-type but used the information theoretic estimates of contextual uncertainty that were updated dynamically, based on what the subject actually observed (see Figure 1A and the Experimental Procedures for details). This approach thus encodes online learning of the order within a sequence, assuming that each event is sampled from a discrete probability distribution.

Our model assumes that subjects model contingencies as being stationary and unchanging within an experimental block. Within the context of the present task, this assumption matches the known generative distribution. The contribution of this paper is to relax the assumption that a subject's response to an event type does not change with experience as is the case in a categorical model. For this, we used a stationary model to learn the probability of event types sequentially within a block, assuming the block structure of the true generative distribution is known. A model that learns transitions between different blocks could be implemented by using a parameterized function to weight past observations, which could take a number of forms including its duration and rate of decay into the past. This will be the subject of future work. However, we illustrate the issue by including two additional models that depend on the maximal window length of past observations from which predictions are based. These were the extreme scenarios of minimal and (near) maximal forgetting. Maximal forgetting would be when the prior is constant for every trial (i.e., it is not updated). If this is chosen to be uniform then every event will be equally likely, i.e. have the same "surprise" throughout the experiment. This meant we compared three models in which the maximal number of past observations were one block, all blocks (i.e., all past observations from previous blocks), and the four most recent trials. Examples of regressors, assuming these different window lengths into the past, are shown in Figure S2.

General Linear Model of Data

To test the hypothesis that surprise and entropy could explain behavioral and physiological responses, we used empirical Bayes to estimate a regression model. There were T trials per subject and S subjects. Data from all subjects was concatenated in a vector, Y, of length $T \times S$. This was fitted using a three-level hierarchical model

$$\begin{aligned} Y &= Z_1 w_1 + e_1 \\ w_1 &= Z_2 w_2 + e_2 \\ w_2 &= e_3 \\ e_1 &\sim N(0, \lambda_1^{-1} I_{TS}) \\ e_2 &\sim N(0, \lambda_2^{-1} I_{PS}) \\ e_3 &\sim N(0, \lambda_3^{-1}) \end{aligned} \tag{5}$$

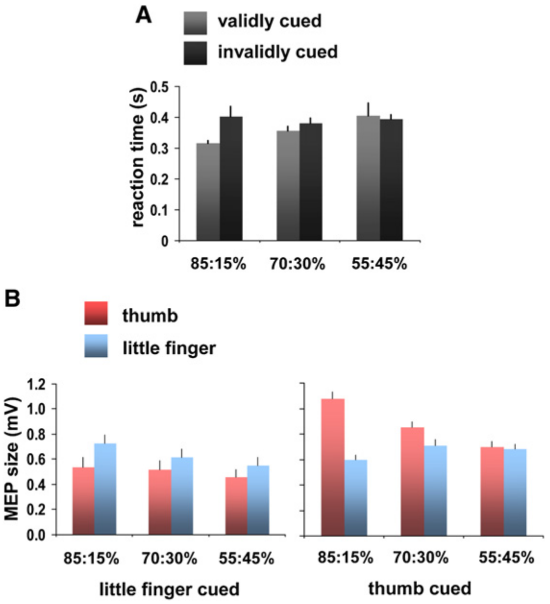

Figure S1. Average Behavioral and Electrophysiological Results

(A) Average reaction times (RTs; mean + SEM) across conditions.

(B) Average changes in corticospinal excitability (mean + SEM), measured from motor-evoked potentials (MEP) in the right thumb (red) and little finger (blue) muscles during preparatory delay periods.

Parameters weights $\{w_1, w_2\}$ scale each column of the design matrices $\{Z_1, Z_2\}$ and hyperparameters $\{\lambda_1, \lambda_2, \lambda_3\}$ control the precision (inverse variance) of noise at each level, $\{e_1, e_2, e_3\}$; these correspond to within-subject error, between-subject error and shrinkage priors on the group-parameters, w_2. The first level design matrix, Z_1 was block diagonal, with dimensions $TS \times PS$, with P regressors per subject. These correspond to subject-specific explanatory variables (i.e., surprise, entropy, error trials, and a constant term). The second design matrix, $Z_2 = 1_S \otimes I_P$, models between-subject differences in the parameter weights, where 1_S was a column of ones of length S and I_P a $P \times P$ identity matrix. Posterior densities over model parameters and hyperparameters were optimized by using standard techniques [S13]. The posterior density represents the degree of belief in the parameter values given data, e.g., reaction time or CSE.

The model evidence $p(y|M_i)$ for the ith model, M_i, was approximated by the marginal likelihood, computed after optimizing the model [S13]. Note that model parameters are integrated out of this expression and therefore include a model complexity term [S13]. This evidence is used to compare competing models defined in terms of the explanatory variables in Z_1. Note that the evidence does not depend on the parameters or their number and, therefore, accounts properly for model complexity when used for Bayesian model comparison. In brief the log evidence comprises two terms; the accuracy of a model or the expected log-likelihood and the complexity, which is a function of the number of and uncertainty about the free parameters. Bayesian model comparison therefore furnishes the most accurate and parsimonious model (see Penny et al., 2004, for details [S14]).

Models were compared using the ratio of the log marginal likelihoods, F_i and F_j, of models i and j. The difference between these two numbers relates to the model evidence ratio as follows

$$F_i - F_j \approx \log\left(\frac{p(y|M_i)}{p(y|M_j)}\right) \quad \Rightarrow \quad \exp(F_i - F_j) \approx \frac{p(y|M_i)}{p(y|M_j)}. \tag{6}$$

This means that a difference of +3 corresponds approximately to 20:1 odds, i.e., exp(3) ≈ 20, in favor of model i over j, whereas -3 corresponds to 20:1 odds in favor of model j. In the present case, positive values reflect stronger evidence in favor of the model containing entropy ($\hat{H}$) and surprise ($\hat{I}$), whereas negative values would indicate stronger evidence for the alternative models tested.

Supplemental References

S1. Wassermann, E.M. (1998). Risk and safety of repetitive transcranial magnetic stimulation: report and suggested guidelines from the International Workshop on the Safety of Repetitive Transcranial Magnetic Stimulation, June 5–7, 1996. Electroencephalogr. Clin. Neurophysiol. *108*, 1–16.

S2. van Elswijk, G., Kleine, B.U., Overeem, S., and Stegeman, D.F. (2007). Expectancy induces dynamic modulation of corticospinal excitability. J. Cogn. Neurosci. *19*, 121–131.

S3. Touge, T., Taylor, J.L., and Rothwell, J.C. (1998). Reduced excitability of the cortico-spinal system during the warning period of a reaction time task. Electroencephalogr. Clin. Neurophysiol. *109*, 489–495.

S4. Hasbroucq, T., Kaneko, H., Akamatsu, M., and Possamai, C.A. (1997). Preparatory inhibition of cortico-spinal excitability: A transcranial magnetic stimulation study in man. Cogn. Brain Res. *5*, 185–192.

S5. Hasbroucq, T., Kaneko, H., Akamatsu, N., and Possamai, C.A. (1999). The time-course of preparatory spinal and cortico-spinal inhibition: An H-reflex and transcranial magnetic stimulation study study in man. Exp. Brain Res. *124*, 33–41.

S6. Mars, R.B., Bestmann, S., Rothwell, J.C., and Haggard, P. (2007). Effects of spatial and motor attention on corticospinal excitability in a delayed-response task. Exp. Brain Res. *182*, 125–129.

S7. Carpenter, R.H. (2004). Contrast, probability, and saccadic latency; evidence for independence of detection and decision. Curr. Biol. *14*, 1576–1580.

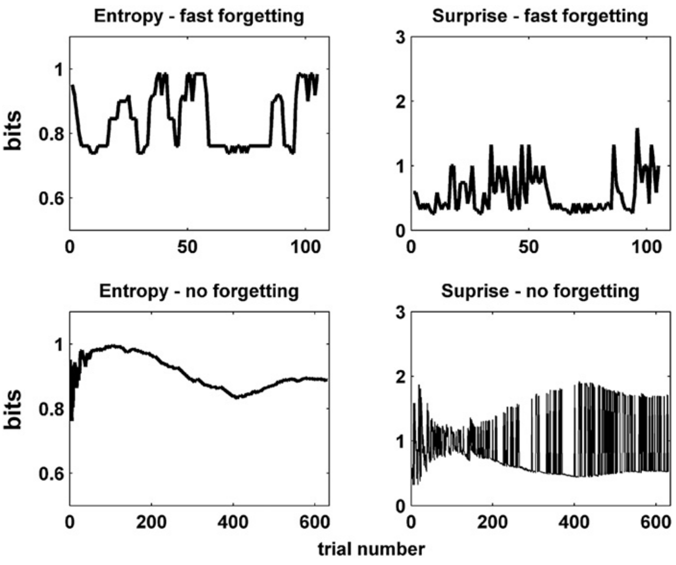

Figure S2. Regressors for Alternative Models

Information theoretic quantities computed from models assuming no forgetting and near maximal forgetting. Top, entropy and surprise from a block sequence generated from a 55%–45% valid-invalid CS block in which only the four most recent previous trials are used. Bottom, entropy and surprise when previous trials from all previous blocks are used. The ensuing time series were used as predictors for modeling corticospinal excitability (CSE) and reactions times (RT) across the entire series of trials of each participant.

S8. Behrens, T.E., Woolrich, M.W., Walton, M.E., and Rushworth, M.F. (2007). Learning the value of information in an uncertain world. Nat. Neurosci. *10*, 1214–1221.

S9. Porrill, J., Frisby, J.P., Adams, W.J., and Buckley, D. (1999). Robust and optimal use of information in stereo vision. Nature *397*, 63–66.

S10. Najemnik, J., and Geisler, W.S. (2005). Optimal eye movement strategies in visual search. Nature *434*, 387–391.

S11. Carpenter, R.H., and Williams, M.L. (1995). Neural computation of log likelihood in control of saccadic eye movements. Nature *377*, 59–62.

S12. Reddi, B.A., Asrress, K.N., and Carpenter, R.H. (2003). Accuracy, information, and response time in a saccadic decision task. J. Neurophysiol. *90*, 3538–3546.

S13. Friston, K., Mattout, J., Trujillo-Barreto, N., Ashburner, J., and Penny, W. (2007). Variational free energy and the Laplace approximation. Neuroimage *34*, 220–234.

S14. Penny, W.D., Stephan, K.E., Mechelli, A., and Friston, K.J. (2004). Comparing dynamic causal models. Neuroimage *22*, 1157–1172.
